# Novel Nomograms Individually Predicting Overall Survival of Non-metastatic Colon Cancer Patients

**DOI:** 10.3389/fonc.2020.00733

**Published:** 2020-05-06

**Authors:** Jun-Peng Pei, Chun-Dong Zhang, Yu Liang, Cheng Zhang, Kun-Zhe Wu, Zhe-Ming Zhao, Dong-Qiu Dai

**Affiliations:** ^1^Department of Gastrointestinal Surgery, The Fourth Affiliated Hospital of China Medical University, Shenyang, China; ^2^Department of Gastrointestinal Surgery, Graduate School of Medicine, University of Tokyo, Tokyo, Japan; ^3^Cancer Center, The Fourth Affiliated Hospital of China Medical University, Shenyang, China

**Keywords:** colon cancer, prognosis, overall survival, nomogram, prediction model

## Abstract

**Background:** This study aimed to develop an effective prognostic nomogram for predicting non-metastatic colon cancer.

**Methods:** The Surveillance, Epidemiology, and End Results program was utilized to analyze patients who underwent surgical therapy (25,350 for training, 10,860 for validation). Nomograms were created depending upon multivariate analysis in the training cohort and were compared to current American Joint Committee on Cancer (AJCC) classifications. Areas under the receiver-operating characteristic curves (AUCs), Akaike's information criterions (AICs), and calibration curves were used. The clinical benefit was measured using decision curve analyses (DCAs). The validation cohort was used to validate the results.

**Results:** Nomogram 1 included age, gender, histological grade, T stage, number of retrieved lymph nodes, tumor size, and N stage. Nomogram 2 included age, gender, histological grade, T stage, number of retrieved lymph nodes, tumor size, and number of positive lymph nodes. The prognostic discrimination of nomogram 1 (AUC, 0.729, 95% CI, 0.723–0.736) was better than that of nomogram 2 (AUC, 0.704, 95% CI, 0.698–0.710, p < 0.001) in five-year overall survival in the training cohort. Nomogram 1 (AIC, 137,319) also showed superior model-fitting compared to nomogram 2 (AIC, 137,453). Similarity, nomogram 1 was better than the AJCC 6th and 8th TNM classifications. DCA revealed that nomogram 1 had a superior net benefit than other models. These findings were validated using the validation cohort.

**Conclusions:** The proposed nomogram 1 was a better prognostic prediction model with better discrimination and superior model-fitting for patients with non-metastatic colon cancer, which might prove to be clinically helpful.

## Introduction

Colon cancer is the third most commonly diagnosed cancer among both males and females in the United States (1). Although some progress has been made in the therapy of colon cancer in the past decades (2, 3), local recurrence and distant metastasis remain a challenge for clinicians (4). The accuracy of survival prediction for patients is critical for postoperative treatment decisions and surveillance. Therefore, tools necessary to provide prognosis for colon cancer patients are critical for helping medical professionals consult and advise patients on their treatment options.

The American Joint Committee on Cancer (AJCC) tumor-node-metastasis (TNM) staging system is the current gold standard for risk assessment (5). It is the most basic and common staging system for evaluating prognosis for colon cancer patients undergoing surgery. For colon cancer patients who are not distantly metastatic, the TNM staging system is determined by two factors: the degree of entry into the intestinal wall and the number of locoregional positive lymph nodes (6). In fact, because of the clinicopathological features and tumor biology variations, the outcomes are quite different, so it is assumed that patients in each group have homogenous results (7). In addition, the classification nature of the TNM staging scheme forces continuous variables into categorical variables, might further limiting prediction accuracy (8). It is increasingly recognized that in addition to the TNM staging system, other clinical factors may contribute significantly to individual predictions of prognosis, such as age, histological type, degree of differentiation, systemic inflammation, and nutritional status (9, 10).

A nomogram is an effective tool for visualizing regression models used to quantify individual risk by including multiple important prognostic factors. It has been shown to achieve a good predictive performance in a variety of cancers. Previous studies in pancreatic cancer (11), uveal melanoma (12), hepatocellular carcinoma (13), and intrahepatic cholangiocarcinoma (14) have shown that nomograms could improve predictive accuracy and provide patients and physicians with a more comprehensive outcome measure when making treatment-related decisions.

In previous studies focusing on colorectal cancer, nomograms were applied to predict overall survival, disease-related survival prognosis, risk of recurrence and metastasis, as well as adjuvant chemotherapy. In the development of the nomograms, some studies used number of positive lymph nodes as a continuous variable (8, 15–18), while others used it as a categorical variable (19–23). However, few studies have applied both the AJCC N stage and number of positive lymph nodes (continuous and categorical variables) to develop nomograms, and to compare their discriminations, model-fittings, and net benefits in predicting overall prognosis.

This study aimed to create a prognostic model of colon cancer depending upon independently prognostic factors of Cox proportional-hazards models. Predictive utility of the nomogram was further compared to the AJCC 6th and 8th TNM staging systems (24). This nomogram is expected to provide more personalized prognostic predictions that will help clinicians and patients make better treatment choices.

## Materials and Methods

### Patients

Using the Surveillance, Epidemiology, and End Results (SEER) program, 691,749 colon cancer patients between 1973 and 2015 were screened (25). The inclusion criteria were as follows: (1) colon cancers from SEER; (2) necessary information available; (3) aged between 18 and 72 years; (4) primary and single tumors; (5) no distant metastasis (M0); (6) received surgery; (7) no preoperative therapy; (8) survival longer than one month; and (9) follow-up ≥ 60 months or until death. Exclusion criteria were as follows: (1) no available information; (2) aged <18 or > 72 years; (3) multiple colon cancers; (4) distant metastasis (M1); (5) no surgical treatment; (6) received preoperative therapy; (7) postoperative survival less than one month; and (8) lost to follow-up or follow-up <60 months. Finally, a total of 36,210 patients were included and randomized into training (70%, *n* = 25,350) and validation cohorts (30%, *n* = 10,860).

### Statistical Analyses

Baseline clinical variable characteristics between training and validation cohorts were compared with Student's *t*-tests or Mann-Whitney *U* tests. Survival curves were depicted using the Kaplan-Meier methods with log-rank tests. Nomograms were developed depending upon prognostic factors of multivariate Cox proportional hazards models.

The predictive discriminations of nomograms and current AJCC TNM classifications were assessed using areas under the receiver-operating characteristic curves (AUCs). The Hanley and McNeil tests were then used to compare the AUCs. The Akaike's information criterion (AIC) (26) and calibration curve (27) were applied to evaluate the nomogram prediction model-fitting. Higher AUCs indicated better discrimination and lower AICs indicated superior model-fitting. The calibration curves were assessed by reviewing the predicted versus actual probabilities. A perfectly accurate classification would result in a calibration curve where most observed and predicted probabilities fall along the 45-degree line. In addition, clinical benefit was measured using decision curve analyses (DCAs) (28, 29).

All data were analyzed using the SPSS 22.0 statistical package (SPSS Inc., Chicago, IL, USA), MedCalc (Version 15.2, Ostend, Belgium), and R version 3.5.6 (http://www.r-project.org/). All tests were two-sided and *p*-values <0.05 were considered statistically significant. The authors signed a data use agreement with SEER. The approval of an institutional review board was not required as the SEER database holds publicly available deidentified data.

## Results

### Clinicopathologic Characteristics

**Figure 1:**
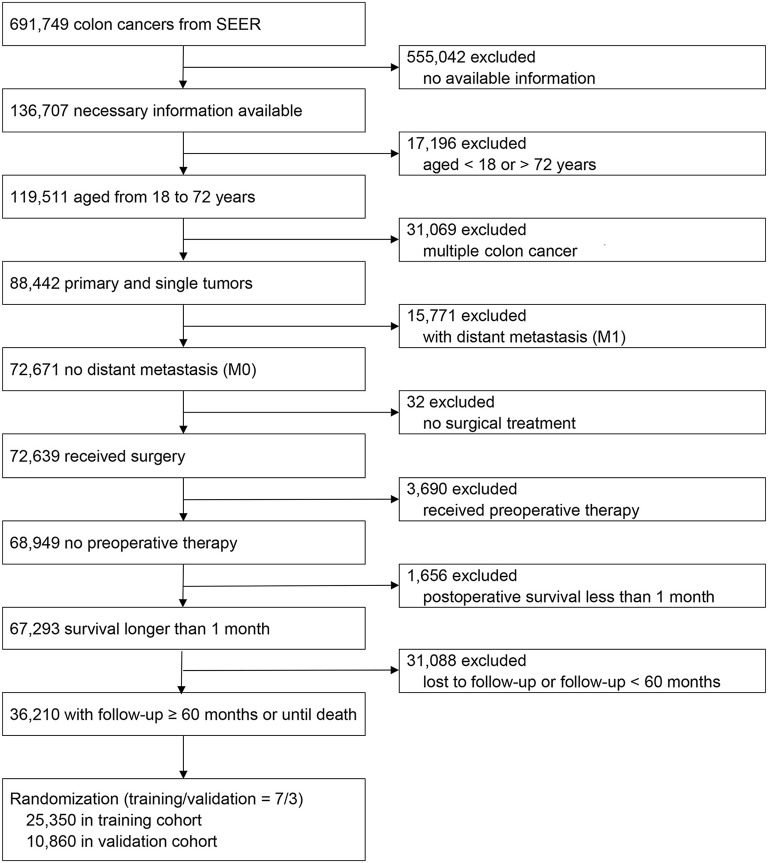
Flow chart for patient selection and study development.

Numbers of patients excluded at each step during patient selection process is shown in Figure 1. Patients were categorized according to the AJCC 6th and 8th TNM staging systems. Demographic and clinical characteristics of the training and validation cohorts are shown in Table 1. Baseline characteristics of the validation cohort were similar to the training cohort (Student' *t*-test or Mann-Whitney *U* test, *p* > 0.05 for all).

**Table 1:** Baseline characteristics of training and validation cohorts.

**Variables**	**Training Cohort^a^*n* = 25,350 (%)**	**Validation Cohort^a^*n* = 10,860 (%)**	***P* value**
Age, years			0.963
Median (range)	60 (18-72)	60 (20-72)	
Gender			0.607
Male	13275 (52.4)	5655 (52.1)	
Female	12075 (47.6)	5205 (47.9)	
Histological grade			0.857
Grade I	2266 (8.9)	999 (9.2)	
Grade II	18404 (72.6)	7856 (72.3)	
Grade III	4273 (16.9)	1815 (16.7)	
Grade IV	407 (1.6)	190 (1.7)	
AJCC 8th T stage			0.281
T1	2640 (10.4)	1077 (9.9)	
T2	4190 (16.5)	1782 (16.4)	
T3	15486 (61.1)	6695 (61.6)	
T4a	1751 (6.9)	763 (7.0)	
T4b	1283 (5.1)	543 (5.0)	
AJCC 8th N stage			0.258
N0	14762 (58.2)	6293 (57.9)	
N1a	3240 (12.8)	1399 (12.9)	
N1b	3352 (13.2)	1398 (12.9)	
N2a	2233 (8.8)	940 (8.7)	
N2b	1763 (7.0)	830 (7.6)	
AJCC 8th TNM stage			0.383
I	5493 (21.7)	2321 (21.4)	
IIA	8011 (31.6)	3456 (31.8)	
IIB	667 (2.6)	260 (2.4)	
IIC	591 (2.3)	256 (2.4)	
IIIA	1150 (4.5)	455 (4.2)	
IIIB	6949 (27.4)	2974 (27.4)	
IIIC	2489 (9.8)	1138 (10.5)	
AJCC 6th TNM stage			0.358
I	5476 (21.6)	2318 (21.3)	
IIA	7971 (31.4)	3445 (31.7)	
IIB	1251 (4.9)	516 (4.8)	
IIIA	1134 (4.5)	448 (4.1)	
IIIB	5577 (22.0)	2381 (21.9)	
IIIC	3941 (15.5)	1752 (16.1)	
Tumor size, mm			0.810
Median (range)	42 (1-150)	41.5 (1-150)	
Positive lymph nodes			0.158
Mean (range)	1.6 (0–59)	1.7 (0–54)	
Examined lymph nodes			0.315
Median (range)	16 (1-89)	16 (1-89)	

*AJCC, American Joint Committee on Cancer*.

a *Ratio of training to validation cohorts is 7:3 by randomized number using R software*.

In the training cohort, the median age was 60 years (range: 18 to 72 years), median tumor size was 42 mm (range: 1 to 150 mm), and median number of retrieved lymph nodes was 16 (range: 1 to 89). A total of 13,275 (52.4%) patients were male and 12,075 (47.6%) were female. According to the AJCC 6th TNM staging system, 5,476 (21.6%) patients were stage I, 7,971 (31.4%) were stage IIA, 1,251 (4.9%) were stage IIB, 1,134 (4.5%) were stage IIIA, 5,577 (22.0%) were stage IIIB, and 3,941 (15.5%) were stage IIIC. According to the AJCC 8th TNM staging system, 5,493 (21.7%) patients were stage I, 8,011 (31.6%) were stage IIA, 667 (2.6%) were stage IIB, 591 (2.3%) were stage IIC, 1,150 (4.5%) were stage IIIA, 6,949 (27.4%) were stage IIIB, and 2,489 (9.8%) were stage IIIC. Univariate analysis identified age, gender, histological grade, AJCC 8th T stage, number of retrieved lymph nodes, tumor size, AJCC 8th N stage, and number of positive lymph nodes significantly associated with overall survival (Table 2). The results were similar in the validation cohort (Supplementary Table S1).

**Table 2:** Univariate analysis of the training cohort.

**Variables**	**3-year OS (%)**	**5-year OS (%)**	**HR (95% CI)**	***P* value**
Age, years	86.0	78.8	1.023 (1.021–1.026)	<0.001
Gender				
Male	84.7	76.7	1 (Ref)	–
Female	87.6	81.1	0.763 (0.728–0.799)	<0.001
Histological grade				
Grade I	91.9	86.7	1 (Ref)	–
Grade II	87.7	80.3	1.399 (1.271–1.539)	<0.001
Grade III	77.1	69.6	2.104 (1.896–2.334)	<0.001
Grade IV	70.9	64.2	2.590 (2.167–3.095)	<0.001
AJCC 8th T stage				
T1	94.5	90.9	1 (Ref)	–
T2	93.8	89.7	1.246 (1.099–1.412)	<0.001
T3	85.9	78.0	2.314 (2.081–2.573)	<0.001
T4a	72.1	61.4	4.278 (3.782–4.840)	<0.001
T4b	63.2	51.8	5.696 (5.021–6.462)	<0.001
AJCC 8th N stage				
N0	91.5	86.2	1 (Ref)	–
N1a	85.8	78.0	1.509 (1.403–1.623)	<0.001
N1b	83.3	73.3	1.884 (1.723–1.974)	<0.001
N2a	75.2	64.1	2.636 (2.454–2.832)	<0.001
N2b	59.7	47.1	4.330 (4.036–4.645)	<0.001
Positive lymph nodes	86.0	78.8	1.096 (1.092–1.100)	<0.001
Tumor size, mm	86.0	78.8	1.003 (1.003–1.004)	<0.001
Retrieved lymph nodes	86.0	78.8	0.989 (0.986–0.991)	<0.001

*AJCC, American Joint Committee on Cancer; CI, confidence interval; HR, hazard ratio; OS, overall survival; Ref, reference*.

### Nomogram 1

**Figure 2:**
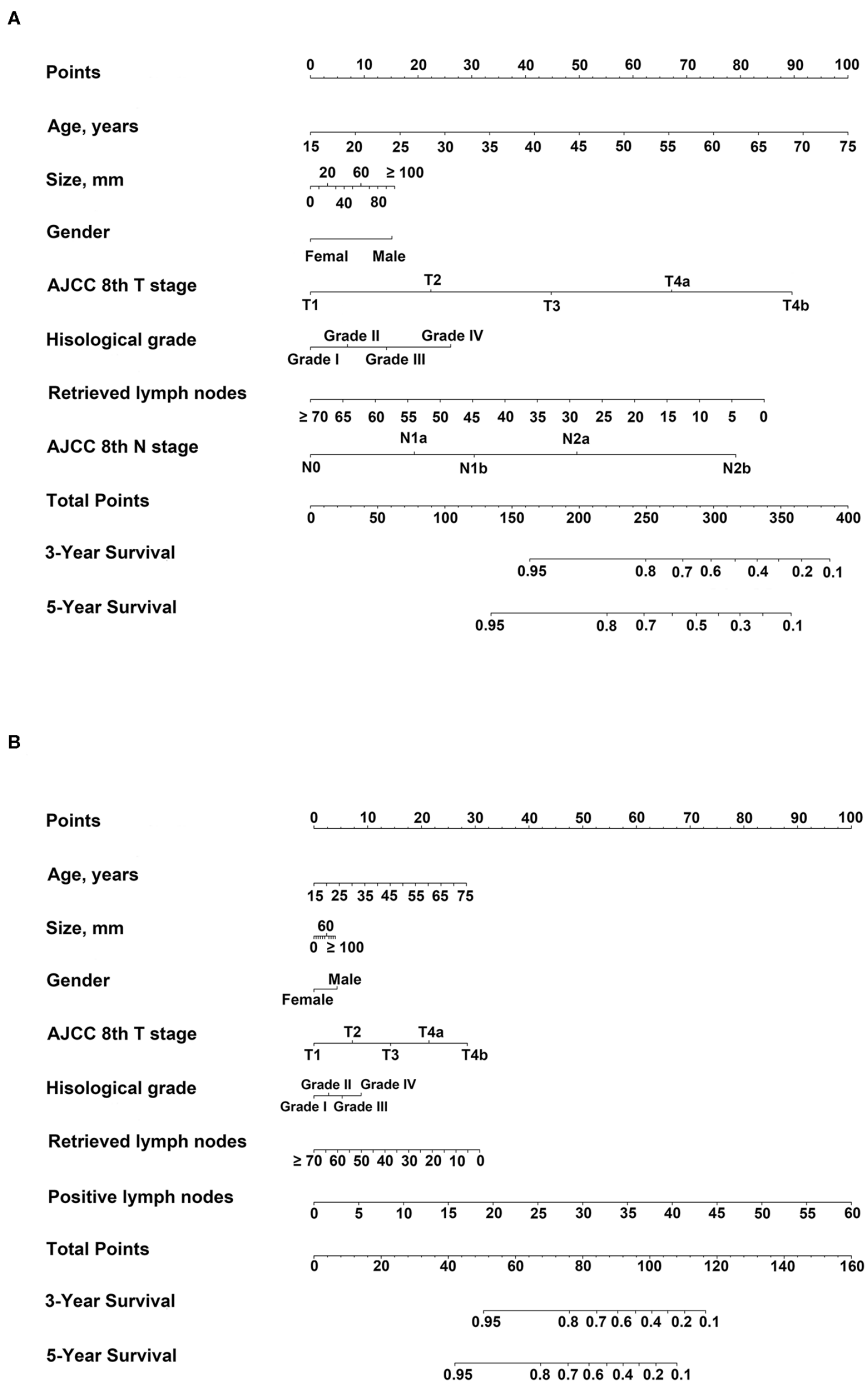
Nomograms predicting three- and five-year overall survival (OS). For each patient, corresponding clinicopathological feature points were calculated and summed up to obtain total points. Predicted three- and five-year OS can be estimated based on total points for each patient. **(A)** Nomogram 1: variables included age, gender, histological grade, AJCC 8th T stage, tumor size, retrieved lymph nodes, and AJCC 8th N stage; **(B)** Nomogram 2: variables included age, gender[[Inline Image]], histological grade, AJCC 8th T stage, tumor size, RLNs, and number of positive lymph nodes.

Depending upon univariate analysis, statistically significant factors including age, gender, histological grade, T stage, number of retrieved lymph nodes, and tumor size were identified as independent prognostic factors and were included in multivariate analysis of Cox proportional hazards models together with the N stage. Significant factors in the multivariate analysis were further incorporated into nomogram 1 (Table 3, Figure 2A). The results were similar in the validation cohort (Supplementary Table S2).

**Table 3:** Multivariable analyses of the training cohort.

**Variables**	**Multivariable analysis 1**		**Multivariable analysis 2**	
	**HR (95% CI)**	***p* value**	**HR (95% CI)**	***P* value**
Age, years	1.029 (1.026–1.031)	<0.001	1.027 (1.025–1.030)	<0.001
Gender				
Male	1 (Ref)	–	1 (Ref)	–
Female	0.772 (0.737–0.810)	<0.001	0.780 (0.744–0.817)	<0.001
Histological grade				
Grade I	1 (Ref)	–	1 (Ref)	–
Grade II	1.143 (1.038–1.259)	0.007	1.177 (1.069–1.296)	0.001
Grade III	1.290 (1.160–1.436)	<0.001	1.358 (1.220–1.511)	<0.001
Grade IV	1.549 (1.319–1.891)	<0.001	1.663 (1.389–1.992)	<0.001
AJCC 8th T stage				
T1	1 (Ref)	–	1 (Ref)	–
T2	1.164 (1.026–1.321)	0.019	1.220 (1.075–1.384)	0.002
T3	1.791 (1.603–2.001)	<0.001	2.056 (1.843–2.294)	<0.001
T4a	2.814 (2.473–3.202)	<0.001	3.330 (2.932–3.783)	<0.001
T4b	4.068 (3.555–4.656)	<0.001	4.563 (3.990–5.217)	<0.001
AJCC 8th N stage				
N0	1 (Ref)	–		
N1a	1.397 (1.297–1.504)	<0.001		
N1b	1.689 (1.575–1.810)	<0.001		
N2a	2.330 (2.164–2.509)	<0.001		
N2b	3.826 (3.549–4.124)	<0.001		
Positive lymph nodes			1.099 (1.094–1.105)	<0.001
Tumor size, mm	1.002 (1.001–1.002)	<0.001	1.001 (1.001–1.002)	<0.001
Retrieved lymph nodes	0.971 (0.978–0.983)	<0.001	0.976 (0.973–0.978)	<0.001

### Nomogram 2

According to univariate analysis, statistically significant factors including age, gender, histological grade, T stage, number of retrieved lymph nodes, and tumor size were included in multivariate analysis of Cox proportional hazards models together with the number of positive lymph nodes. Significant factors in the multivariate analysis were further incorporated into nomogram 2 (Table 3, Figure 2B). The results were similar in the validation cohort (Supplementary Table S2).

### Three- and Five-Year Overall Survival of AJCC 6th and 8th Staging Systems

**Figure 3:**
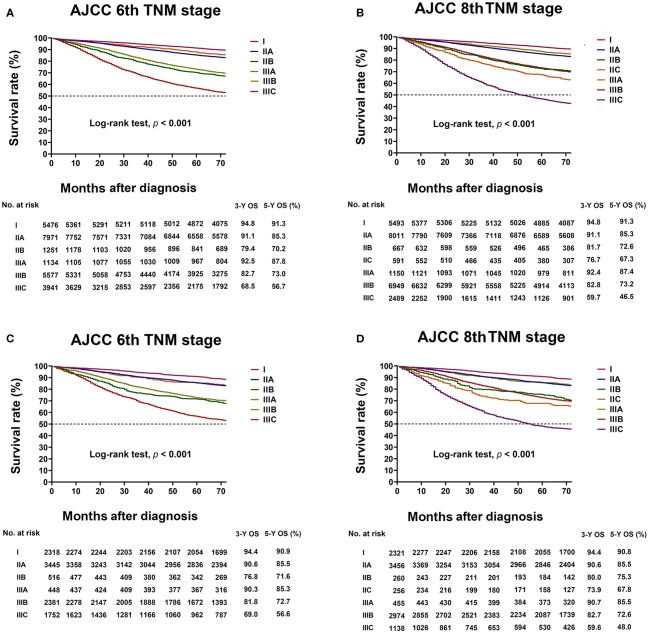
Kaplan–Meier survival curve based on the AJCC 6th TNM and AJCC 8th TNM classifications. **(A)** Kaplan–Meier survival curves based on AJCC 6th TNM classification in the training cohort. **(B)** Kaplan–Meier survival curves based on AJCC 8th TNM classification in the training cohort. **(C)** Kaplan–Meier survival curves based on AJCC 6th TNM classification in the validation cohort. **(D)** Kaplan–Meier survival curves based on AJCC 8th TNM classification in the validation cohort.

In the training cohort analyzed with the AJCC 6th TNM staging system, the three- and five-year Overall Survival (OS) of stages IIA, IIB, and IIIA was 91.1 and 85.3%, 79.4 and 70.2%, and 92.5 and 87.8%, respectively. The prognosis of stage IIIA was better than that of stages IIA and IIB (Log-rank test, *p* < 0.05 for all, Figure 3A). The results were similar in the validation cohort (Figure 3C).

In the 8th TNM staging system, the three- and five-year OS of stages IIA, IIB, IIC, and IIIA was 91.1 and 85.3%, 81.7 and 72.6%, 76.7 and 67.3%, and 92.4 and 87.4%, respectively. The prognosis of Stage IIIA was better than that of stages IIA, IIB, and IIC (Log-rank test, *p* < 0.05 for all, Figure 3B). The results were similar in the validation cohort (Figure 3D).

### Comparison of Predictive Performance Between two Nomograms

**Figure 4:**
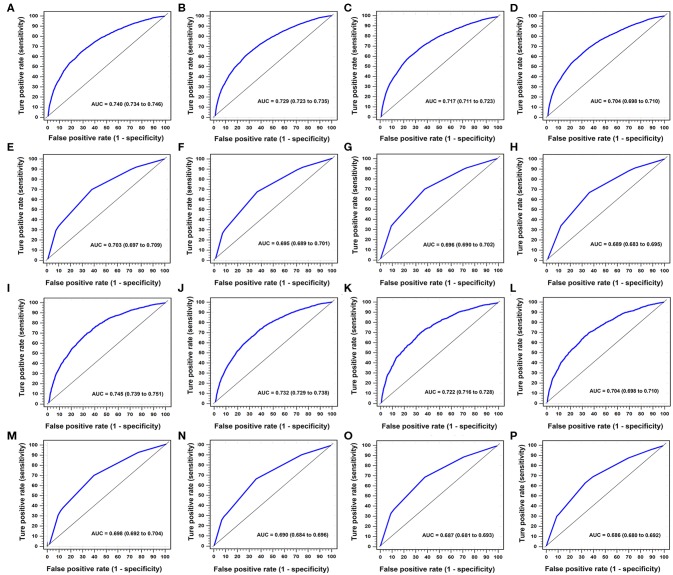
Time-dependent areas under receiver-operating characteristic (ROC) curves (AUCs) in training and validation cohorts for three- and five-year OS. In training cohort, **(A, B)** were nomogram 1 for three- and five-year OS, respectively. **(C, D)** were nomogram 2 for three- and five-year OS, respectively. **(E, F)** were AJCC 8th TNM classification for three- and five-year OS, respectively. **(G, H)** were AJCC 6th TNM classification for three- and five-year OS, respectively. In validation cohort, **(I, J)** were nomogram 1 for three- and five-year OS, respectively. **(K, L)** were nomogram 2 in three- and five-year OS, respectively. **(M, N)** were AJCC 8th TNM classification for three- and five-year OS, respectively. **(O, P)** were AJCC 6th TNM classification for three- and five-year OS, respectively.

In the training cohort, AUCs of nomogram 1 at three- and five-year OS were 0.740 (95% CI, 0.734–0.746) and 0.729 (95% CI, 0.723–0.736), respectively, with AIC for OS of 137,319 (Table 4, Figures 4A,B). AUCs of nomogram 2 at three- and five-year OS were 0.717 (95% CI, 0.711–0.723) and 0.704 (95% CI, 0.698–0.710), respectively, with AIC for OS of 137,453 (Table 4, Figures 4C,D).

**Table 4:** Comparisons of different predictive models of nomograms with AJCC 6th and 8th TNM classifications.

	**Training cohort**			**Validation cohort**		
	**3-year OS AUC (95% CI)**	**5-year OS AUC (95% CI)**	**AIC**	**3-year OS AUC (95% CI)**	**5-year OS AUC (95% CI)**	**AIC**
Nomogram 1	0.740 (0.734–0.746)	0.729 (0.723–0.736)	137319	0.745 (0.739–0.751)	0.732 (0.729–0.738)	54121
Nomogram 2	0.717 (0.711–0.723)	0.704 (0.698–0.710)	137453	0.722 (0.716–0.728)	0.704 (0.698–0.710)	54170
AJCC 6th TNM	0.696 (0.690–0.702)	0.689 (0.683–0.695)	138717	0.687 (0.681–0.693)	0.686 (0.680–0.692)	54731
AJCC 8th TNM	0.703 (0.697–0.709)	0.695 (0.689–0.701)	138404	0.698 (0.692–0.704)	0.690 (0.684–0.696)	54628
*P* value^a^	<0.001	<0.001	–	<0.001	<0.001	–
*P* value^b^	<0.001	<0.001	–	<0.001	<0.001	–
*P* value^c^	<0.001	<0.001	–	<0.001	<0.001	–
*P* value^d^	<0.001	<0.001	–	<0.001	<0.001	–
*P* value^e^	<0.001	<0.001	–	<0.001	<0.001	–
*P* value^f^	<0.001	<0.001	–	<0.001	<0.001	–

*A higher AUC indicates better discrimination and a lower AIC indicates superior model-fitting*.

*Nomogram 1, variables included, age, gender, histological grade, AJCC 8th T stage, tumor size, retrieved lymph nodes, and AJCC 8th N stage*.

*Nomogram 2, variables included, age, gender, histological grade, AJCC 8th T stage, tumor size, retrieved lymph nodes, and number of positive lymph nodes*.

Hanley & McNeil tests of AUCs:

a *Nomogram 1 vs. Nomogram 2*;

b *Nomogram 1 vs. AJCC 6th TNM*;

c *Nomogram 1 vs. AJCC 8th TNM*.

d *Nomogram 2 vs. AJCC 6th TNM*;

e *Nomogram 2 vs. AJCC 8th TNM*;

f *AJCC 6th TNM vs. AJCC 8th TNM*.

In the validation cohort, AUCs of nomogram 1 at three- and five-year OS were 0.745 (95% CI, 0.739–0.751) and 0.732 (95% CI, 0.729–0.738), respectively, with the AIC for OS of 54,121 (Table 4, Figures 4I,J). AUCs of nomogram 2 at three- and five-year OS were 0.722 (95% CI, 0.716–0.728) and 0.704 (95% CI, 0.698–0.710), respectively, with the AIC for OS of 54,170 (Table 4, Figures 4K,L).

**Figure 5:**
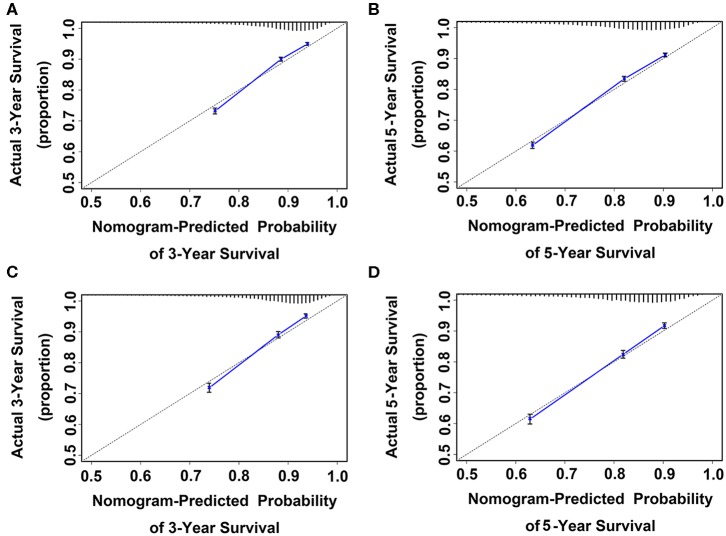
Calibration curve for predicting patient survival at **(A)** three and **(B)** five years in the training cohort and at **(C)** three and **(D)** five years in the validation cohort. Nomogram-predicted probability of OS is plotted on the x-axis; actual OS is plotted on the y-axis. Shorter distance between two curves indicates higher accuracy.

In the training cohort, the prognostic discrimination of nomogram 1 was better than of nomogram 2 (Hanley and McNeil test, all *p* < 0.001, Table 4) in three- and five-year OS. Nomogram 1 also showed a superior model-fitting compared to nomogram 2 according to the AICs and calibration curves (Table 4, Figures 5A–D). Similar findings were validated in the validation cohort.

### Comparison of Predictive Performance Between AJCC 6th and 8th TNM Staging Systems

In the training cohort, the AUCs for the AJCC 8th TNM staging system for three- and five-year OS were 0.703 (95% CI, 0.697–0.709) and 0.695 (95% CI, 0.689–0.701), respectively, with the AIC for OS of 138,404 (Table 4, Figures 4E,F). The AUCs for the AJCC 6th TNM staging system at three- and five-year OS were 0.696, (95% CI, 0.690–0.702) and 0.689 (95% CI, 0.683–0.695), respectively, with the AIC for OS of 138,717 (Table 4, Figures 4G,H).

In the validation cohort, the AUCs for the AJCC 8th classification for three- and five-year OS were 0.698 (95% CI, 0.692–0.704) and 0.690 (95% CI, 0.684–0.696), respectively, with the AIC for OS of 54,628 (Table 4, Figures 4M,N). The AUCs for the AJCC 6th classification at three- and five-year OS were 0.687, (95% CI, 0.681–0.693) and 0.686, (95% CI, 0.680–0.692), respectively, with the AIC for OS of 54,731(Table 4, Figures 4O,P).

In the training cohort, the prognostic discrimination of the AJCC 8th classification was better than of the AJCC 6th classification (Hanley and McNeil test, all *p* < 0.001, Table 4) in three- and five-year OS. The AJCC 8th classification also showed superior model-fitting compared to the AJCC 6th classification according to the AICs (Table 4). Similar findings were validated in the validation cohort.

### Comparison of Predictive Performance Between Nomogram 1 and AJCC 8th TNM Staging Systems

In the training cohort, the prognostic discrimination of nomogram 1 was better than the AJCC 8th classification (Hanley and McNeil test, all *p* < 0.001, Table 4) in three- and five-year OS in the two cohorts. Nomogram 1 also showed superior model-fitting compared to the AJCC 8th classification according to the AICs in the two cohorts (Table 4). Similar findings were validated in the validation cohort.

### Comparison of Clinical Usefulness Between Nomograms and AJCC TNM Staging System Using Decision Curve Analyses

**Figure 6:**
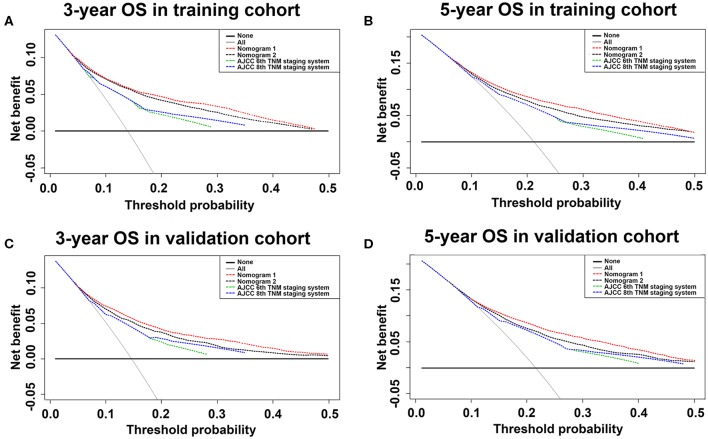
Decision curve analysis of training and validation cohorts for three- and five-year OS. Decision curve analysis was used to compare clinical net benefits between nomograms and conventional staging systems in terms of three-year OS for **(A)** training and **(C)** validation cohorts and five-year OS for **(B)** training and **(D)** validation cohorts. For decision curve analysis, horizontal solid black line assumed no patients would die and dotted gray line assumed all patients would die.

Using the decision curve analyses (DCAs) for both training and validation cohorts, nomogram 1 showed better net benefits with wider ranges of threshold probabilities and improved performance than nomogram 2 and AJCC 6th and 8th TNM staging systems for predicting three- and five-year OS in colon cancer patients (Figures 6A–D). These results represent a superior estimation of decision outcomes at higher threshold probability levels.

## Discussion

Accurate predictions of the prognosis for colon cancer patients are critical for further postoperative treatment and follow-up planning. Traditionally, the survival outcome of postoperative colon cancer patients is predicted based on the AJCC TNM staging system. Since the 1940s, the TNM staging system has determined the extent of cancer based only on the depth of tumor infiltration and the number of positive lymph nodes. Revisions of the staging system were modified every six to eight years and, until recently, it has been considered the most comprehensive tool for predicting the prognosis and predictive grouping of colon cancer patients. However, when the AJCC 6th TNM staging system was released in 2002 (24), its accuracy was questioned because the survival rate of patients with stage IIIA was superior to that of patients with stage IIB colon cancer (30). The AJCC 7th edition released in 2010 (31) has been staging more accurately than the AJCC 6th edition for improving the prognosis. However, the AJCC 7th edition has not eliminated survival discrepancies between stages II and IIIA colon cancers. The AJCC 8th edition (6) released in 2017 showed no changes in stages I–III compared to the AJCC 7th TNM staging system. A similar issue was observed in the current study, where the AJCC 6th staging system did not satisfactorily stratify patients between stages II and III. Patient prognosis with stage IIIA was better than that with stage II. The 8th TNM staging system made the staging more elaborate compared to the 6th TNM classification. However, it still does not do a good job at stratifying patients between stages II and III.

In this study, prognostic nomograms based on the results of the Cox proportional-hazards model were developed and validated to predict survival probabilities in patients undergoing surgery for non-metastatic colon cancer. Compared to the 6th and 8th editions of the AJCC staging system based on the depth of infiltration and the number of positive lymph nodes, a nomogram can integrate various prognostic factors to make more personalized predictions for patients. Age, gender, histological grade, T stage, number of retrieved lymph nodes, tumor size, N stage, and number of positive lymph nodes were integrated into the nomogram. Many researchers have also shown that these clinicopathological factors are associated with the prognosis of colon cancer patients (32). It should also be noted that the number of retrieved lymph nodes was an independent factor in the prognosis of colon cancer. Many previous studies have shown that it is also an independent prognostic factor for many other malignancies and the larger number of lymph nodes removed meant a better survival prognosis (33, 34). Perhaps the most important reason is that as the lymph nodes are more extensively removed, more potentially positive lymph nodes will not be missed, providing enough positive lymph nodes to be used for precise staging.

In the establishment of the nomogram, some researchers have used the number of positive lymph nodes as a continuous variable (8, 15–18), while others used it as a categorical variable (19–23). Few researchers have used both the AJCC N stage and number of positive lymph nodes as variables to develop nomograms and to compare their accuracy in predicting prognosis in their studies. The present study developed a nomogram using these variables and evaluated the accuracy in predicting prognosis. The results showed that the nomogram including the AJCC 8th N stage had a better survival prediction accuracy than the nomogram including the number of positive lymph nodes. The nomogram incorporates clinically common pathological factors and provides a more personalized prognostic prediction than the AJCC staging systems. In addition, nomograms have better clinical benefits and other researchers have achieved the same results in other oncology studies. Through this novel and easy-to-implement scoring system, personalized survival prognosis predictions after surgery can be easily obtained. Identifying colon cancer patients with different survival risks based on the nomograms may have an impact on further treatment or follow-up plans.

Using the SEER (25) data allows to draw reasonable conclusions consistent with general clinical practice based on a large sample number of colon cancer patients, which is impossible to achieve in a single institutional study. However, this study had some limitations that should be concerning. First, even if the SEER database was regularly checked for discrepancies, it has been reported that its accuracy is 98% and the possibility of incorrect coding or erroneous data still exists. In addition, other potentially prognostic factors including lymphatic vessel invasion, marginal status, surgical procedures, postoperative complications, laboratory indices, and chemotherapy data were not used. More well-known predictors for improving model performance should be applied. Besides, the current study was limited by its retrospective nature, although it was based on a large database. Furthermore, the currents study was based on a Western database of SEER program (25, 35), and further cohorts from Eastern countries are still needed to validate our findings.

## Conclusions

In summary, this study developed a prognostic nomogram for patients with non-metastatic colon cancer. The nomogram improves the estimates provided by the current AJCC 8th TNM staging system and can more accurately estimate the survival rate for individual patients after surgery. It might be useful for medical professionals to develop further treatment options and long-term follow-up plans for patients undergoing colon cancer surgery.

## Data Availability Statement

The datasets analyzed during this current study are available in SEER database (https://seer.cancer.gov/) to extract the eligible cases. The data are also available from the corresponding author on reasonable request.

## Ethics Statement

Ethical approval was not needed as 3rd party data from the SEER database was used.

## Author Contributions

J-PP, C-DZ, and D-QD conceived and designed the study. J-PP and YL analyzed the data. J-PP, C-DZ, CZ, and K-ZW wrote the paper. D-QD and Z-MZ reviewed the draft. All authors read and approved the final manuscript.

## Conflict of Interest

The authors declare that the research was conducted in the absence of any commercial or financial relationships that could be construed as a potential conflict of interest.
