# Supplementary material for: Novel Nomograms Individually Predicting Overall Survival of Non-metastatic Colon Cancer Patients
Source: Front Oncol. 2020 May 6;10:733. doi: 10.3389/fonc.2020.00733 (PMC7218119; doi:10.3389/fonc.2020.00733)
Supplement: Supplementary file 1 [file Table_1.DOCX]

Supplementary Material

| TABLE S1. Univariate analysis of the validation cohort. | | | | |
| --- | --- | --- | --- | --- |
| Variables | 3-year OS (%) | 5-year OS (%) | Hazard ratio (95% CI) | *P* value |
| Age, years | 85.3 | 78.5 | 1.022 (1.019–1.025) | <0.001 |
| Gender |  |  |  |  |
| Male | 84.7 | 76.7 | 1 (Ref) | – |
| Female | 86.8 | 80.5 | 0.781 (0.746–0.819) | <0.001 |
| Histological grade |  |  |  |  |
| Grade I | 91.0 | 85.6 | 1 (Ref) | – |
| Grade II | 87.5 | 80.2 | 1.330 (1.212–1.460) | <0.001 |
| Grade III | 76.4 | 68.7 | 2.053 (1.855–2.272) | <0.001 |
| Grade IV | 71.2 | 64.2 | 2.528 (2.121–3.012) | <0.001 |
| AJCC 8th T stage |  |  |  |  |
| T1 | 94.6 | 91.2 | 1 (Ref) | – |
| T2 | 93.5 | 89.3 | 1.240 (1.093–1.407) | <0.001 |
| T3 | 85.7 | 77.7 | 2.319 (2.084–2.581) | <0.001 |
| T4a | 71.5 | 61.2 | 4.354 (3.849–4.926) | <0.001 |
| T4b | 62.3 | 51.5 | 5.655 (4.982–6.420) | <0.001 |
| AJCC 8th N stage |  |  |  |  |
| N0 | 90.9 | 86.3 | 1 (Ref) | – |
| N1a | 86.7 | 79.5 | 1.401 (1.250–1.570) | <0.001 |
| N1b | 79.9 | 70.3 | 2.057 (1.858–2.278) | <0.001 |
| N2a | 77.1 | 63.3 | 2.639 (2.363–2.947) | <0.001 |
| N2b | 59.7 | 48.9 | 4.080 (3.674–4.530) | <0.001 |
| Positive lymph nodes | 85.3 | 78.5 | 1.096 (1.092–1.099) | <0.001 |
| Tumor size, mm | 85.3 | 78.5 | 1.007 (1.006–1.008) | <0.001 |
| Retrieved lymph nodes | 85.3 | 78.5 | 0.988 (0.986–0.991) | <0.001 |
| AJCC, American Joint Committee on Cancer; CI, confidence interval; OS, overall survivalr. | | | | |

| TABLE S2. Multivariable analyses of the validation cohort. | | | | | |
| --- | --- | --- | --- | --- | --- |
| Variables | Multivariable analysis 1 | |  | Multivariable analysis 2 | |
|  | Hazard ratio (95% CI) | *P* value |  | Hazard ratio (95% CI) | *P* value |
| Age, years | 1.029 (1.025–1.033) | <0.001 |  | 1.027 (1.023–1.031) | <0.001 |
| Gender |  |  |  |  |  |
| Male | 1 (Ref) | – |  | 1 (Ref) | – |
| Female | 0.851 (0.793–0.914) | <0.001 |  | 0.852 (0.794–0.915) | <0.001 |
| Histological grade |  |  |  |  |  |
| Grade I | 1 (Ref) | – |  | 1 (Ref) | – |
| Grade II | 1.094 (0.949–1.262) | 0.215 |  | 1.126 (0.977–1.298) | 0.102 |
| Grade III | 1.278 (1.091–1.497) | 0.002 |  | 1.326 (1.132–1.553) | <0.001 |
| Grade IV | 1.549 (1.189–2.017) | 0.001 |  | 1.592 (1.223–2.074) | 0.001 |
| AJCC 8th T stage |  |  |  |  |  |
| T1 | 1 (Ref) | – |  | 1 (Ref) | – |
| T2 | 1.286 (1.055–1.566) | 0.013 |  | 1.339 (1.099–1.630) | 0.004 |
| T3 | 1.923 (1.613–2.293) | <0.001 |  | 2.208 (1.856–2.627) | <0.001 |
| T4a | 2.929 (2.390–3.589) | <0.001 |  | 3.519 (2.880–4.300) | <0.001 |
| T4b | 4.174 (3.378–5.157) | <0.001 |  | 4.813 (3.901–5.938) | <0.001 |
| AJCC 8th N stage |  |  |  |  |  |
| N0 | 1 (Ref) | – |  |  |  |
| N1a | 1.304 (1.162–1.464) | <0.001 |  |  |  |
| N1b | 1.873 (1.688–2.079) | <0.001 |  |  |  |
| N2a | 2.329 (2.077–2.611) | <0.001 |  |  |  |
| N2b | 3.691 (3.299–4.129) | <0.001 |  |  |  |
| Positive lymph nodes |  |  |  | 1.100 (1.093–1.108) | <0.001 |
| Tumor size, mm | 1.001 (1.000–1.002) | 0.005 |  | 1.001 (1.000–1.002) | 0.017 |
| Retrieved lymph nodes | 0.977 (0.973–0.981) | <0.001 |  | 0.971 (0.967–0.976) | <0.001 |
| AJCC, American Joint Committee on Cancer; CI, confidence interval; OS, overall survival.  Multivariable analysis 1, variables included, age, gender, histological grade, AJCC 8th T stage, tumor size, retrieved lymph nodes, and AJCC 8th N stage;  Multivariable analysis 2, variables included, age, gender, histological grade, AJCC 8th T stage, tumor size, retrieved lymph nodes, and positive lymph nodes. | | | | | |

**
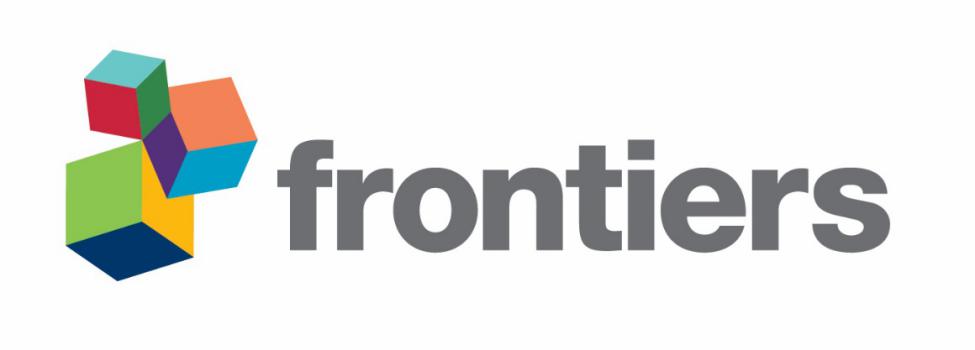
**
